# Non-destructive, spectrophotometric analysis of the thickness of the cell-multilayered periosteal sheet

**DOI:** 10.1186/s40729-022-00419-1

**Published:** 2022-05-02

**Authors:** Hachidai Aizawa, Takashi Uematsu, Atsushi Sato, Hideo Masuki, Hideo Kawabata, Tetsuhiro Tsujino, Kazushige Isobe, Yutaka Kitamura, Masaki Nagata, Koh Nakata, Tomoyuki Kawase

**Affiliations:** 1 Tokyo Plastic Dental Society, Kita-ku, Tokyo, Japan; 2 Division of Pioneering Advanced Therapeutics, Niigata University Hospital, Niigata, Japan; 3 Division of Oral Bioengineering, Institute of Medicine and Dentistry, Niigata University, Niigata, Japan

**Keywords:** Periosteal cell sheet, Multilayers, Thickness, Spectrophotometric analysis, Quality assurance

## Abstract

**Background:**

Autologous tissue-engineered periosteal sheets, which have been clinically applied for periodontal regeneration, sinus lift, and alveolar ridge augmentation, are enriched with osteoblast precursor cells and the abundant deposition of collagen type I in the extracellular spaces. Their quality is inspected prior to clinical use; however, most criteria cannot be evaluated without sacrificing samples. To reduce such losses, we developed a non-destructive optical method that can quantitatively evaluate the thickness of the periosteal sheet.

**Methods:**

Dispersed periosteal cells were inoculated into small pieces of collagen sponge (Terudermis®) and plated into 60-mm dishes for further explant culture using a conventional medium and a stem-cell culture medium. The thickness of periosteal sheets was evaluated using inverted microscopic, histological, labeling (CellVue®)-based imaging and spectrophotometric (Spectro-1®) methods.

**Results:**

The three-dimensional growth of periosteal sheets did not necessarily correlate with two-dimensional growth. The periosteal sheet prepared with the stem-cell medium formed cell multilayers, a phenomenon that could be observed qualitatively by inverted microscopy. The spectrophotometric analysis enabled the quantitative evaluation of the thickness of the cell multilayer without sacrificing the samples processed for scheduled cell therapy.

**Conclusions:**

The growth of periosteal sheets is influenced by several major factors, including the basic quality of the individual original periosteal tissue segments, the technical expertise of doctors and operators involved in tissue harvesting and processing, and culture conditions. This newly developed spectrophotometric analysis can quantify the thickness of cell-multilayered periosteal sheets for quality assurance in a non-destructive manner, thereby contributing to better bone augmentation prior to implant therapy.

## Introduction

The autologous tissue-engineered periosteal sheet is a promising biomaterial that can be chemically induced to express osteogenic, osteoconductive, and osteoinductive potentials, for bone regenerative therapy [1–4]. In our university hospital, since 2005, this regenerative therapy has been applied in more than 130 cases, such as those of periodontal regeneration and alveolar ridge augmentation, and substantial clinical outcomes have been confirmed without significant complications or adverse effects. With regard to the biological characteristics, the periosteal sheet consists of proliferative immature precursor cells in the osteoblast lineage and abundant deposition of extracellular matrices (ECMs), such as type 1 collagen. Therefore, such cells are embedded in the collagen matrix and form a mechanically tough structure of the cell multilayers. In response to osteoblast induction, cells differentiate into osteogenic cells to produce mineral deposits in culture [4–8]. In animal grafting experiments, periosteal sheets were demonstrated to be capable of inducing osteoclasts [6, 9]. These findings validate the biomedical functions and support the clinical application of periosteal sheets.

In clinical applications, in general, cell medicinal products, e.g., tissue-engineered cells, are required to be assured of their quality regarding viability, purity, identity, potency, stability for the intended use, tumorigenicity, and so on [10]. However, it is difficult to examine the quality of periosteal sheets using these criteria. With regard to the purity, although the major cell contents of the periosteal sheet are osteoblast precursors in the case of the periosteal sheet, cells related to blood capillaries and fibroblastic cells, which do not differentiate from osteogenic cells, are also included. Thus, unlike major cell medicinal products for cell therapy, the periosteal sheet could be categorized into a heterogeneous material (i.e., heterogeneous cell population) rather than a homogeneous one, and the purity could be excluded from the criteria for quality inspection. With regard to potency, which is usually examined by sampling inspection, even though cells in the osteoblast lineage are the major “factors” responsible for osteogenesis, owing to their carrier function for latent transforming growth factor β [11] and other growth factors, the ECM abundantly deposited in the cell-multilayered structure is expected to significantly potentiate the osteogenic function. In addition, the ECM is expected to function as a platform for biomineralization [12].

A practical matter remains to be resolved. The number of periosteal sheets was limited by the size and quality of the harvested periosteal tissue. The periosteal tissue is cut into small pieces and subjected to explant culture [4]. Unlike dispersed cells, periosteal cells cannot be passaged or freely expanded in explant cultures. Thus, the number of periosteal tissue pieces was defined as the number of periosteal sheets when the processing was successfully performed [4, 7, 9]. This practical background suggests that the quality inspection requiring the sacrifice of samples should be limited to secure the number of samples for clinical use unless surgical invasion is extended to harvest larger periosteal tissues than usual ones.

As mentioned earlier, a major characteristic of the periosteal sheet is the cell-multilayered structure. To date, we have performed microscopic and macroscopic observations to check cell multilayers. This method is non-destructive but qualitative, and the results can be influenced by possible biases by inspectors. Thus, convenient methods that can quickly and non-destructively quantify cell multilayers have been required for routine quality assurance. To satisfy these two requirements, in this study, we focused on a spectrophotometric technology and developed a new non-destructive, nonlabelled method using a compact, portable, Bluetooth-enabled, tablet (or smartphone)-operated spectrophotometer.

## Materials and methods

The preparation of tissue-engineered periosteal sheets using a collagen sponge.

**Fig. 1:**
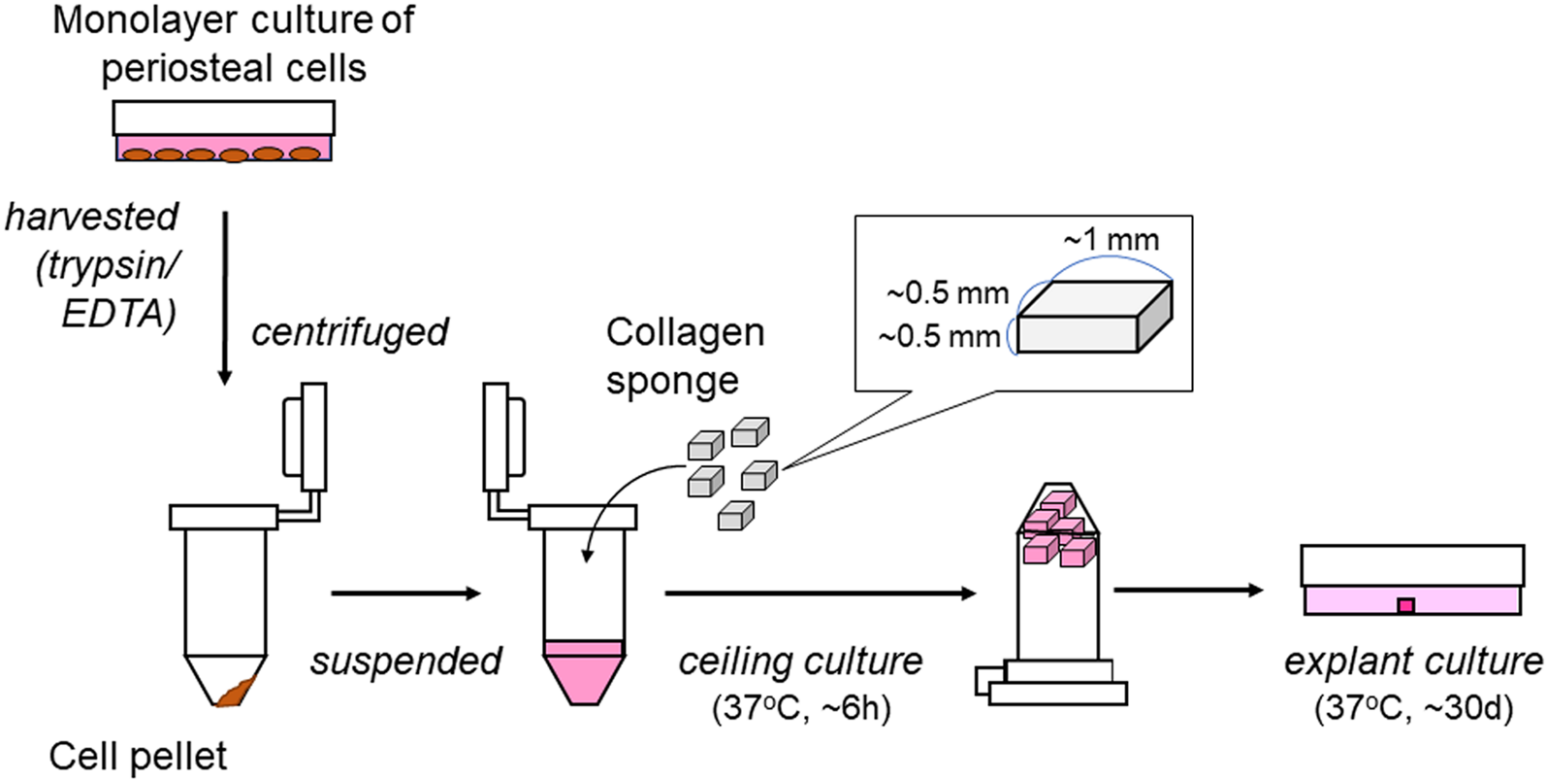
Workflow diagram for the preparation of periosteal sheets using collagen sponge

Periosteal cells were originally expanded in explant cultures of periosteal sheets as described previously [4] and dispersed by enzymatic digestion to be stored in liquid nitrogen. After thawing, the cells were expanded and subjected to the preparation of periosteal sheets. As illustrated in Fig. 1, cells were dispersed in Dulbecco’s modified Eagle’s medium (DMEM) (FUJIFILM Wako Pure Chemical Co., Osaka, Japan) supplemented with 10% fetal bovine serum (FBS) (Thermo Fisher Scientific, Tokyo, Japan), inoculated into small pieces (approximately 0.5 × 1 × 1 mm) of collagen sponge (Terudermis®; Olympus Terumo Biomaterials, Tokyo, Japan), and incubated for approximately 6 h by ceiling culture in a CO_2_ incubator (Sanyo Medical Co., Osaka, Japan). Thereafter, pieces of collagen sponge inoculated with cells were placed at the center of a 60-mm culture dish and subjected to explant cultured in DMEM supplemented with 10% FBS (the conventional medium) or MSC–PCM® medium (Kohjin Bio, Sakado, Japan), which was originally developed for the maintenance of mesenchymal stem cells and modified for explant culture of the periosteal sheet, supplemented with 5% FBS (the stem cell medium) for up to 30 days. Both culture media were supplemented with 25 μg/mL ascorbic acid 2-phosphate (FUJIFILM Wako Pure Chemical Co.) and antibiotics (Thermo Fisher). To prevent or attenuate wave-induced shear stress that detaches collagen sponges from the bottom of the dish, the volume of culture media was limited to 2 mL in the initial period until uniform cell outgrowth and gradually increased up to 4 mL with days of culture. The medium was exchanged every 3 days.

### Evaluation of the growth of the periosteal sheet and cell numbers

With regard to the two-dimensional (2D) growth of periosteal sheets, the edge of periosteal sheets was optically visualized and traced through the illumination of ceiling lights. Diameters were measured at two points (that is, major and minor axes), and the averages were recorded.

With regard to cell numbers, periosteal sheets were rinsed twice with phosphate-buffered saline (PBS) and treated with 0.53 mM ethylenediaminetetraacetic acid and 0.05% trypsin (FUJIFILM Wako Pure Chemical Co.) for 20 min in a CO_2_ incubator. Detached cells were further pipetted into a uniform cell suspension and counted using an automated cell counter (MOXI Z Mini; ORFLO, Ketchum, ID, USA). When collagen matrix fragments were observed in the case of prolonged cultures in the stem cell medium, after mincing with scissors and well pipetting, cell numbers were counted to minimize cell loss.

### Spectrophotometric analysis of the thickness of periosteal sheets

**Fig. 2:**
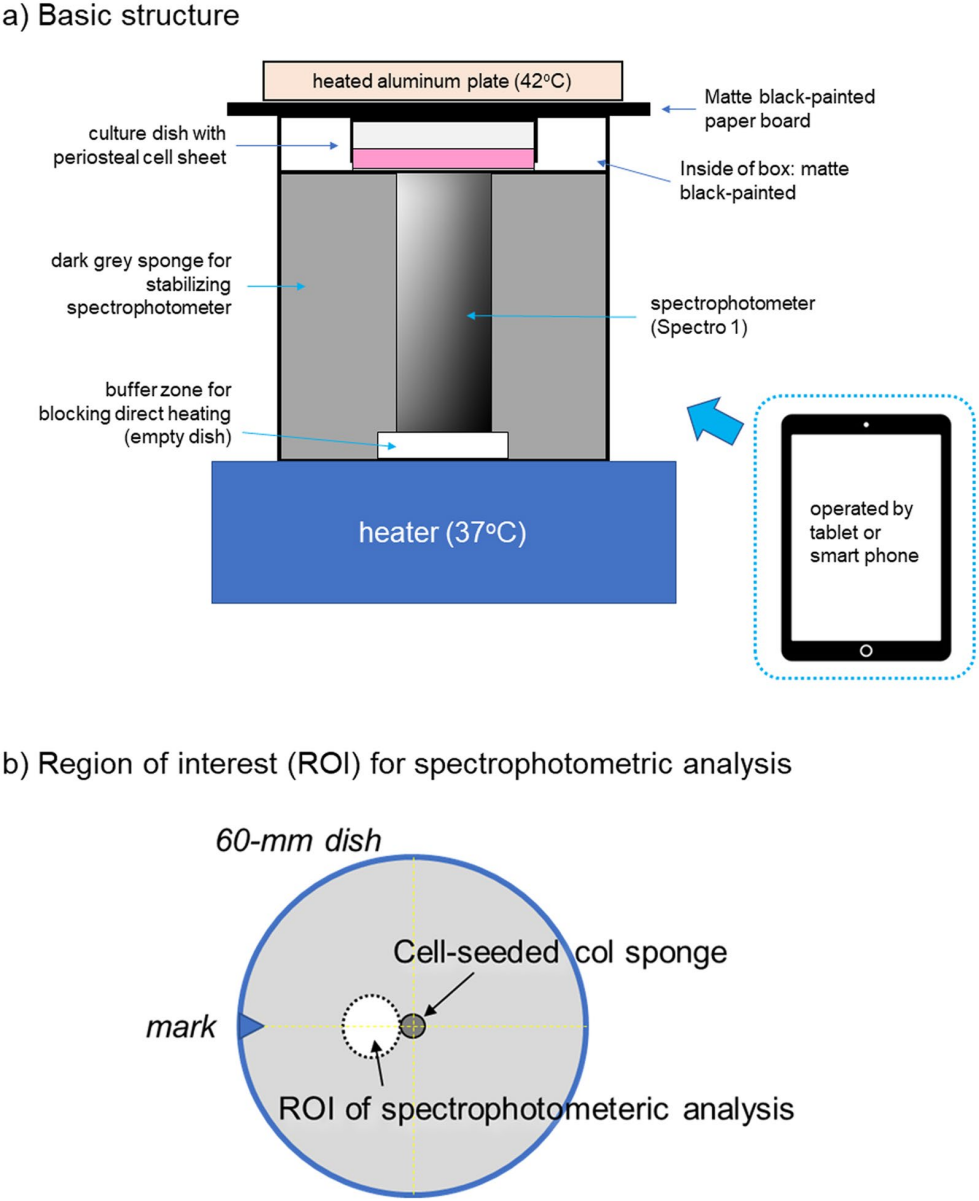
Schema for **a** spectrophotometric device assembly and **b** region of interest for the spectrophotometric analysis

To measure the absorbance of periosteal sheets, a spectrophotometer (Spectro 1®; VARIABLE, Chattanooga, TN, USA) was assembled with a heater, heated aluminum plate, and dark grey sponge (cushion), and a box inside the box was painted black (Fig. 2a). After prewarming, the spectrophotometer was calibrated and subjected to the determination of absorbance was determined.

In the spectrophotometric analysis, generally in the field of biochemistry, transparent liquids are measured mainly by transmitted light. In contrast, these objects, especially thick periosteal sheets, are not liquid and are hardly transmitted by visible light.

This portable spectrophotometer acquires spectra not by transmitted light but by reflected light produced by a white, full-spectrum light-emitting diode [13, 14]. Light reflected from the sample surface was captured by a CMOS digital camera, and sequences of spectral bands were generated and recorded [15]. Therefore, the absorbance values of the complete DMEM and the complete stem cell medium stored at 4 °C (pH approximately 7.4) were 0.020 and 0.007 (arbitrary unit), respectively, at 620 nm of the transmission spectrophotometer using micro cuvettes (SmartSpec Plus®; Bio-Rad, Hercules, CA, USA) (that is, conventional > stem cell medium). In contrast, those of the cell-free media warmed at 37 °C in dishes (pH approximately 7.4) were 0.05854 and 0.06060 at 620 nm of the reflectance spectrophotometer (Spectro 1®) (that is, conventional < stem cell medium). Thus, for appropriate interpretation of the data, it should be noted that the absorbance decreases with the addition of phenol red and possibly similar, but unidentified, minor components of the media in the reflectance spectrophotometer.

The region of interest (ROI) of the spectrophotometric analysis was a spot area (8 mm in diameter) adjacent to the cell-inoculated collagen sponge (Fig. 2b). To reproducibly access the same ROI, a mark was placed on the edge of the dish.

Initially, to determine which wavelength is suitable for following changes in the thickness, spectra of early and prolonged cultures were compared, and 620 nm was adopted.

### Histological examination

Periosteal sheets were detached using a scraper in 10% formaldehyde in 0.1 M phosphate buffer (pH 7.4) for 4 h at 4 °C and fixed between nylon mesh membranes for 6 h, as described previously [4, 7]. Fixed samples were dehydrated, embedded in paraffin, and sectioned sagittally at a thickness of 6 μm. The sections were stained with hematoxylin and eosin (H&E).

### Cell labeling and in vivo near-infrared (NIR) imaging

Periosteal sheets were labeled with CellVue® NIR815 Fluorescent Cell Labeling Kit (LI-COR, Inc., Lincoln, NE, USA) in accordance with the manufacturer's instructions. In brief, adherent periosteal sheets were washed twice with PBS and treated for 5 min at room temperature with the dye diluted in Diluent C at a final concentration of 1 μM [7]. Periosteal sheets were then rinsed twice with PBS and incubated overnight in individual culture media in a CO_2_ incubator prior to examination. According to the manufacturer’s instructions provided by the vendor, we performed NIR imaging using a Pearl® Imager (LI-COR, Inc.), for which excitation/emission wavelengths were fixed at and at 785/820 nm (800 nm channel).

### Statistical analysis

Continuous data are expressed as the mean ± standard deviation of four independent cultures. For two-group comparisons, the nonparametric, two-tailed Mann–Whitney *U* test was performed to compare mean values (SigmaPlot 13.0; Systat Software, Inc., San Jose, CA, USA). Statistical significance was set at *p* < 0.05.

## Results

**Fig. 3:**
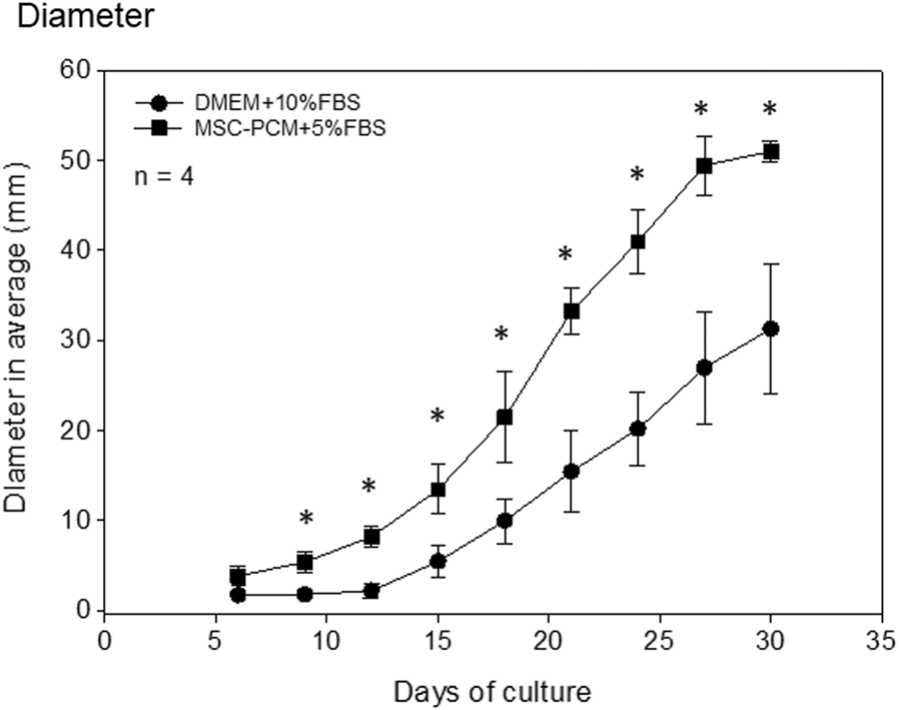
Time-course changes in the diameter on of the average, that is two-dimensional growth of periosteal sheets. *n* = 4. *P < 0.05 vs. DMEM + 10%FBS at the same timepoints

To confirm the structural similarity of the collagen-based periosteal sheet, 2D growth and thick cell multilayers around the collagen sponge were examined. Figure 3 shows the average time-course changes in diameter. Compared with the genuine periosteal sheet that uses periosteal tissue segments [4, 16], cell outgrowth occurred much faster within 3 days of culture, and periosteal sheets grew slightly fast in the conventional medium. The stem cell medium significantly facilitated 2D growth and reached the edge of 60-mm dishes within 30 days.

**Fig. 4:**
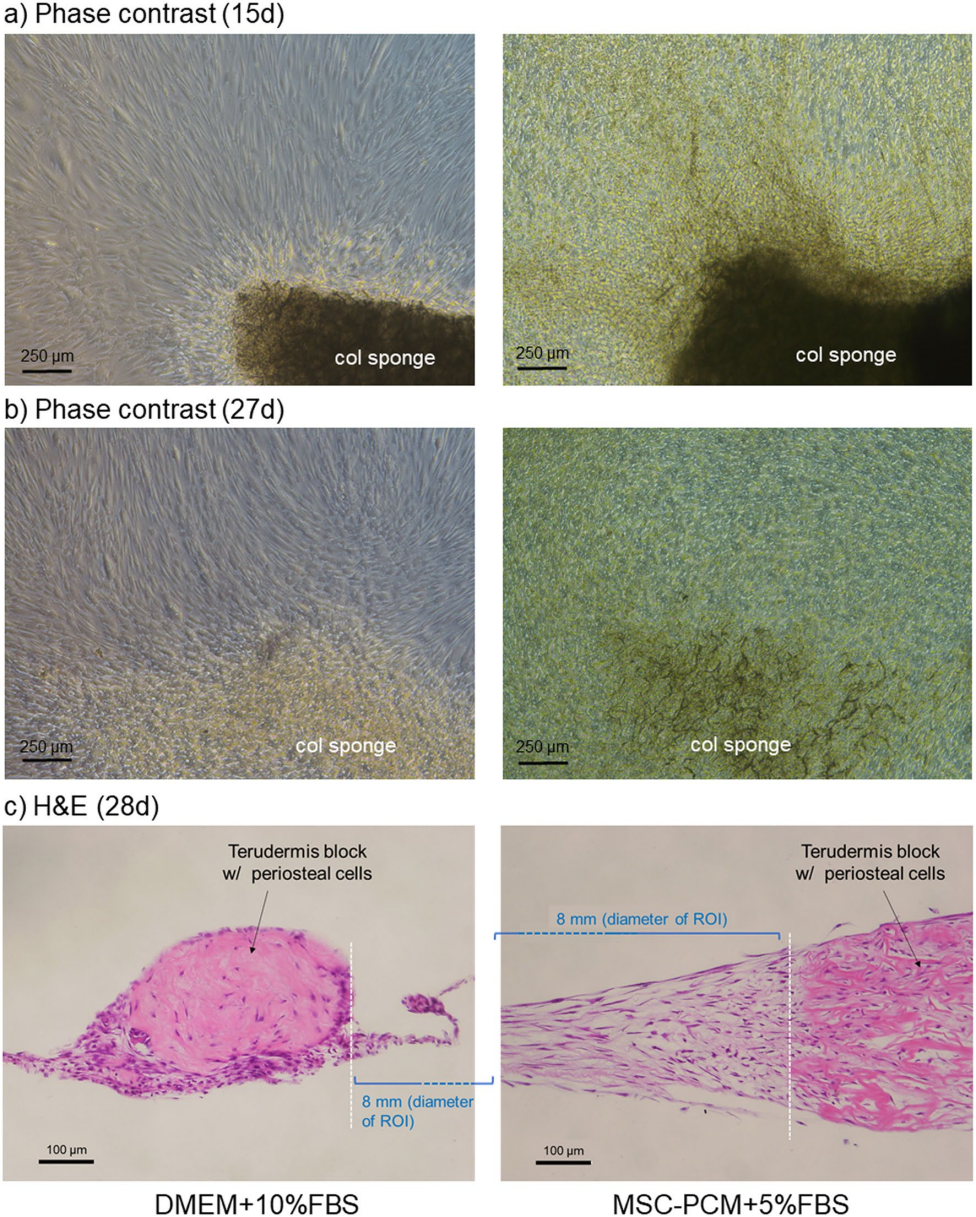
Photomicrographic observations of cell layers formed by cell outgrowths of nearby pieces of a collagen (col) sponge. **a**, **b** Phase-contrast microscopic observations. **c** H&E staining of sagittal sections. The region of interest for the determination of absorbance is indicated by parentheses and blue letters

Figure 4a, b shows photomicrographs of the phase-contrast microscope. In cultures using the conventional medium, cell layers adjacent to the collagen sponge seemed minimal and did not become thick with increasing time of culture. It is noted that the collagen sponge was gradually digested probably by cellular collagenases. In contrast, in the stem cell medium, periosteal sheets at the ROI were much thicker than those in the conventional medium at 15 days. Further cultivation digested collagen sponge and seemed to increase the thickness and integrated cell multilayers and collagen sponges to form a “hybrid membrane.” Fig. 4c supports these findings and shows cell multilayers adjacent to the collagen sponges were much thicker in the stem cell medium than those in the conventional medium: the thickness was 350–400 nm at maximum, seemingly more than 10 layers, in the case of the stem cell medium.

**Fig. 5:**
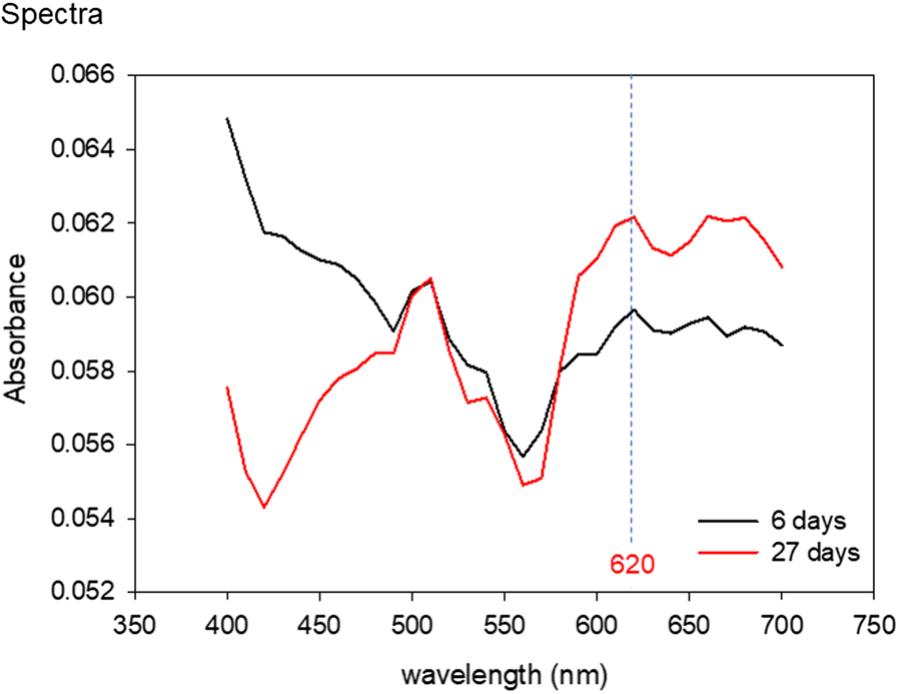
Spectra of early and later cultures of periosteal sheets. These spectra are representative of four pairs of independent cultures

Figure 5 shows the absorbance spectra of the distinguishable periosteal sheets. In the ROI shown in Figs. 2b and 4c, absorbance spectra were collected from early and prolonged cultures of periosteal sheets in the stem cell medium. In the range of 400–500 nm, the absorbance was higher in early cultures than in prolonged cultures, whereas in the range over 570 nm, the order of absorbance magnitude was reversed and the peaks were observed at 620, 660, and 680 nm. Since bacteria and platelet counts are preferably monitored at 600–620 nm [17], the wavelength for monitoring was fixed at 620 nm in the following experiments.

**Fig. 6:**
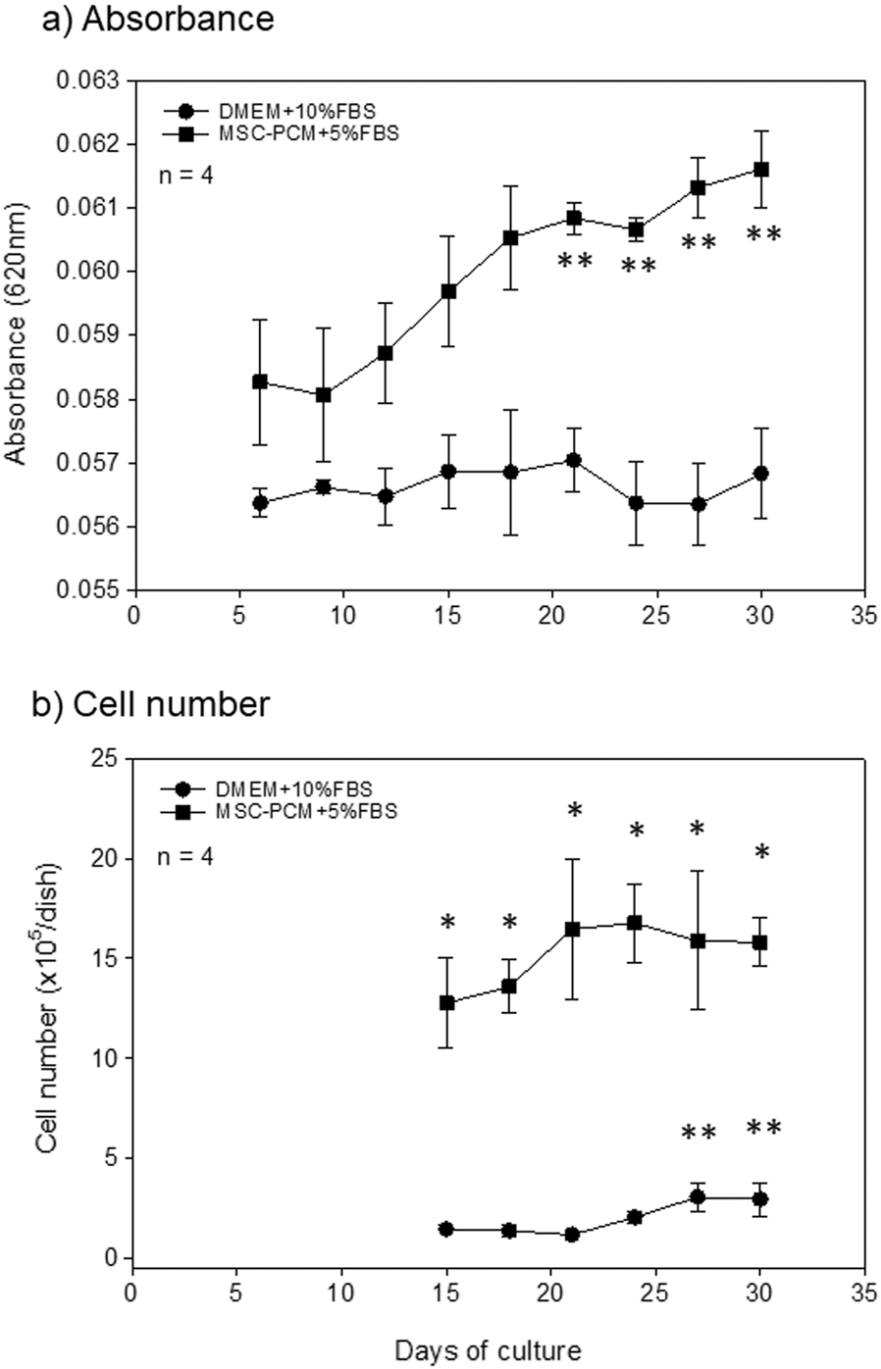
Time-course changes in **a** absorbance values and **b** cell numbers. *n* = 4. *P < 0.05 vs. DMEM + 10%FBS at the same timepoints. **P < 0.05 vs. the same culture conditions at 6 days (absorbance) or 15 days (cell number)

Figure 6a shows the time-course changes in the absorbance of periosteal sheets in the conventional and stem cell media. In the conventional medium, the absorbance was maintained at similar levels throughout the observation period. In contrast, the absorbance in the stem cell medium was higher than that in the conventional medium in the absence of cells and increased with time from 12 days of culture. Significant differences (compared with early culture in the same medium) were observed at 21 days and later. Figure 6b shows the time-course changes in cell numbers in the conventional and stem cell media. At 20 days and later, cell numbers gradually increased and reached maximum levels at 27 days in the conventional medium. In contrast, cell numbers increased and reached maximum levels after 21 days. These cell numbers of the stem-cell medium were consistently greater than those of the conventional medium; however, no significant differences were observed among the cultures in the same stem cell medium.

**Fig. 7:**
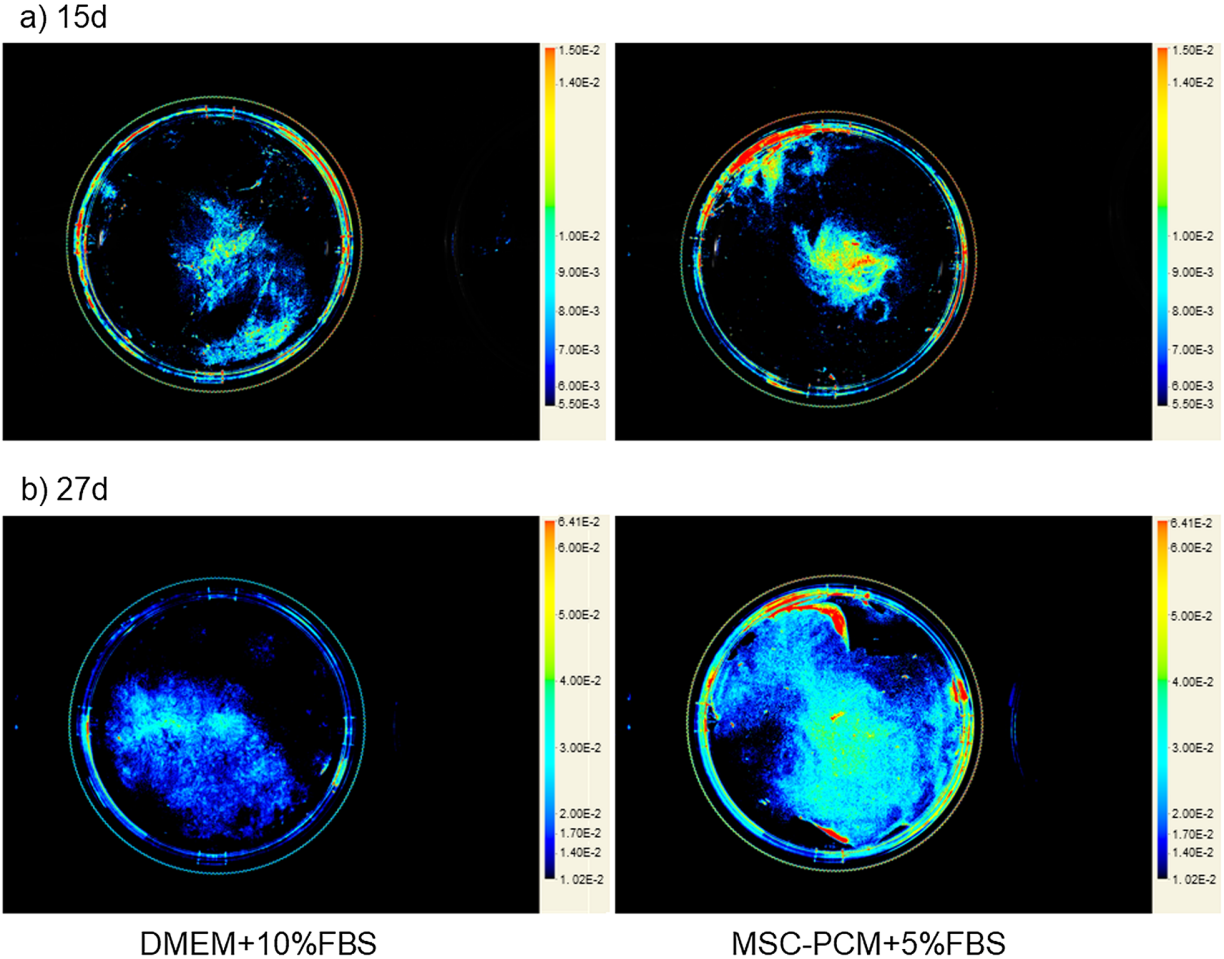
Optical near-infrared imaging of the thickness, that is, three-dimensional growth of living periosteal sheets using CellVue® NIR815

Figure 7 shows the visualization of cell density. To validate the data obtained by the spectrophotometric analysis, cells in periosteal sheets were labeled with the NIR dye and “contour maps” of the 3D growth of periosteal sheets. As observed in the genuine periosteal sheet [7], periosteal sheets cultured in the stem cell medium formed thick cell layers regardless of the culture period. These findings support the data of the spectrophotometric analysis shown in Fig. 6.

## Discussion

The purpose of this study was to develop a method that quantitatively determines the thickness of the periosteal sheet prior to clinical use. It could be expressed as the “identification” of this cell medicinal product. Furthermore, this determination should be performed without destruction, labeling, or any other processing that requires the sacrifice of samples.

The main achievement of this study is the finding that the spectrophotometric analysis at 620 nm could provide quantitative data on the thickness non-destructively without sacrificing the samples. To date, the thickness has been quantified mainly using conventional destructive methods, e.g., histochemical methods. Thus, to avoid loss of samples and waste of time, the thickness has usually been checked qualitatively and intuitively by a macroscopic or microscopic method. This newly developed method provides an easy way to quantify the thickness of the cell-multilayered periosteal sheet, and it could be applied to various thick biological samples regardless of the inclusion of cells with minor modification and optimization.

The primary practical advantage of this method is the quick, non-destructive acquisition of objective data. Prior to clinical use, processed cells are usually subjected to quality inspections to ensure their sterility, purity, identity, potency, tumorigenicity, and stability [10]. Because many inspections require the extraction or fixation of samples, several samples are processed for quality inspection and not for clinical use. Due to the limitation of the explant culture method, a quality inspection cannot be enriched without increasing surgical invasion for the initial periosteum tissue harvest. Thus, non-destructive quantitative methods are required. The present method meets these requirements and is superior to the conventional methods in terms of non-destruction, convenience, speed of analysis, space for installation, and cost of purchase and maintenance. In this study, we collected data from an ROI per dish; however, if available and acceptable, it would be accurate to choose several ROIs per dish to evaluate the overall growth of each whole-periosteal sheet.

The scientific limitations of this method should be discussed. First, since this method is influenced by optical transparency, cell debris, collagen–digestion products, FBS-derived fatty contents, contaminated bacteria, and any other insoluble materials may alter the absorbance. Second, phenol red, a pH indicator, and several other molecules with absorbance at 620 nm may cooperatively increase the absorbance [18] in the transmission type of the spectrophotometer. Phenol red was supplemented in DMEM approximately 2.5-times as much as in the PSC–PCM® medium (15.00 vs. 6.08 mg/L) [19]. In addition, the absorbance may be more or less influenced by the volume of media. To stabilize collagen sponges at the center of dishes, we initially limited the volume of media to approximately 2 mL per dish and increased the volume in a stepwise manner depending on the level of cell outgrowth (that is, prolonged > early culture). However, the absorbance of periosteal sheets cultured in the conventional medium did not increase over time (that is, late = early culture). Thus, we concluded that the volume of the medium did not significantly influence the absorbance.

If more reproducible data on the thickness are required, to exclude possible interfering factors, we would suggest that a protocol composed of gentle rinsing and subsequent temporal replacement of phenol red-free, Ca^2+^, Mg^2+^-containing buffers during the measurement may improve the quantification.

## Clinical relevance

Autologous bone graft is considered as the gold standard in bone regenerative therapy; however, it is strongly associated with postoperative complications and increased surgical costs [20]. To overcome these limitations and, more importantly, to reduce bone graft harvest, we developed a periosteal-sheet therapy [3]. In this therapy, the periosteal sheet is used as a substitute for autologous crushed bone, but not as a barrier membrane or periosteum tissue-like material. Therefore, the periosteal sheet is expected to function as an osteogenic and osteoinductive material. Thus, in general, greater the number of cells and ECMs that are expanded from the limited size of the harvested periosteal tissue within limited time periods, better the quality of the periosteal sheet for clinical use.

The success of implant therapy depends on bone quality and healing at the implant–bone interface [21]; the former determines bone strength and fragility and influences the primary implant stability, while the latter is thought to influence secondary implant stability [22]. Furthermore, the primary bone strength, also known as “bone stiffness,” reflects the stiff, hard mineral crystals of hydroxyapatite. However, its ultimate strength and toughness are believed to depend on an intact network, that is, well-developed crosslinking of type I collagen, which contributes 90% of the bone organic matrix [23, 24].

Type I collagen is also involved in mediating the signaling cascade through interaction with a specific integrin α2β1 for the expression of a mature osteoblast phenotype and mineralization of the ECM [25]. In bone, collagen, as a three-dimensional structure of ECM, acts not only on osteoblast-lineage cells to support cell adhesion, proliferation, and responses to growth factors and differentiation but also on osteoclasts. As a result of all these actions, the bone is newly formed, matured, and resorbed [26]. In addition, bone ECM traps growth factors released by osteoblasts and controls their release [27]. Based on these functional properties, various types of ECM-based scaffolds, such as ECM-modified biomaterial scaffolds and decellularized ECM scaffolds, have been developed and increasingly applied in bone tissue engineering [26, 28].

In our university hospital, the newly modified periosteal-sheet therapy using a thicker periosteal sheet prepared with stem-cell medium was introduced in 2020, and till date, has been applied in 13 clinical cases (vs. the original protocol: 120 cases). Although severe complications or adverse events have been observed, a much longer follow-up period of a sufficient number of patients is needed to carefully evaluate the possible relationship between membrane thickness and clinical outcomes. To evaluate such a possible relationship more precisely, it is necessary to continuously use this technique to determine the thickness of each periosteal sheet upon shipping.

## Conclusions

This newly developed method could be a promising tool for the quantitative analysis of the thickness of cell-multilayered periosteal sheets in a non-destructive manner. Despite several scientific weaknesses, this method is superior to conventional methods from a practical point of view and can be applied to other biological specimens.

## Data Availability

The data are available from the corresponding author on reasonable request.

## Abbreviations

col: Collagen
ROI: Region of interest
ECM: Extracellular matrix
DMEM: Dulbecco’s modified Eagle’s medium
FBS: Fetal bovine serum
2D: Two-dimensional
3D: Three-dimensional
PBS: Phosphate-buffered saline
NIR: Near-infrared
H&E: Hematoxylin and eosin
